# Activation of TLR9 signaling suppresses the immunomodulating functions of CD55^lo^ fibroblastic reticular cells during bacterial peritonitis

**DOI:** 10.3389/fimmu.2024.1337384

**Published:** 2024-05-17

**Authors:** Ting Jiang, Yiming Li, Xingping Huang, Preethi Jayakumar, Timothy R. Billiar, Meihong Deng

**Affiliations:** ^1^ Department of Surgery, University of Pittsburgh, Pittsburgh, PA, United States; ^2^ Institute of Molecular Medicine, Feinstein Institutes for Medical Research, New York, NY, United States; ^3^ Departments of Molecular Medicine and Surgery, Zucker School of Medicine at Hofstra University/Northwell, New York, NY, United States

**Keywords:** fibroblastic reticular cells, fat-associated lymphoid cluster, TLR9, peritoneal immunity, peritonitis

## Abstract

Fibroblastic reticular cells (FRCs) are a subpopulation of stromal cells modulating the immune environments in health and disease. We have previously shown that activation of TLR9 signaling in FRC in fat-associated lymphoid clusters (FALC) regulate peritoneal immunity via suppressing immune cell recruitment and peritoneal resident macrophage (PRM) retention. However, FRCs are heterogeneous across tissues and organs. The functions of each FRC subset and the regulation of TLR9 in distinct FRC subsets are unknown. Here, we confirmed that specific deletion of TLR9 in FRC improved bacterial clearance and survival during peritoneal infection. Furthermore, using single-cell RNA sequencing, we found two subsets of FRCs (CD55^hi^ and CD55^lo^) in the mesenteric FALC. The CD55^hi^ FRCs were enriched in gene expression related to extracellular matrix formation. The CD55^lo^ FRCs were enriched in gene expression related to immune response. Interestingly, we found that TLR9 is dominantly expressed in the CD55^lo^ subset. Activation of TLR9 signaling suppressed proliferation, cytokine production, and retinoid metabolism in the CD55^lo^ FRC, but not CD55^hi^ FRC. Notably, we found that adoptive transfer of *Tlr9* ^-/–^CD55^lo^ FRC from mesenteric FALC more effectively improved the survival during peritonitis compared with WT-FRC or *Tlr9* ^-/–^CD55^hi^ FRC. Furthermore, we identified CD55^hi^ and CD55^lo^ subsets in human adipose tissue-derived FRC and confirmed the suppressive effect of TLR9 on the proliferation and cytokine production in the CD55^lo^ subset. Therefore, inhibition of TLR9 in the CD55^lo^ FRCs from adipose tissue could be a useful strategy to improve the therapeutic efficacy of FRC-based therapy for peritonitis.

## 1 Introduction

Fibroblastic reticular cells (FRCs), characterized as CD45^–^, CD31^–^, Pdgfra^+^, and podoplanin^+^ (PDPN^+^), are a unique subpopulation of fibroblast in lymphoid organs, such as lymph nodes and fat-associated lymphoid clusters (FALCs) in the omentum and mesenteric adipose tissue in the peritoneal cavity (1–3). In the classic paradigm, fibroblasts are considered non-immune cells, providing a structural scaffold for forming lymphoid organs (3). Recently, we and others have shown that FRCs can produce a variety of cytokines, chemokines, and metabolites, such as CXCL13, IL6, and retinoid acid (RA), to modulate immune responses during homeostasis and host defense (1–6). These studies challenge the paradigm and indicate that FRCs play critical immunomodulatory roles in health and diseases.

Emerging studies reveal that fibroblasts are highly heterogeneous. Diverse subsets of FRC have been identified in the spleen (7), lymph node (LN) (8), intestine (9), and synovial tissue (10), where these cells regulate immune surveillance. With the advancement in single-cell technologies, a recent study has constructed fibroblast atlases by integrating mouse and human single-cell transcriptomic data from more than 200,000 fibroblasts across 17 tissues, 50 datasets, and 11 disease states (11). More than 10 subsets of fibroblasts have been identified across organs in health and disease. However, the functions of these FRC subsets, especially the immunomodulatory functions, within distinct organs and tissues, such as the FALC, remain unclear.

We and others have shown that FRCs in lymph nodes and FALC orchestrate peritoneal immunity during abdominal infections. Adoptive transfer of FRC can improve outcomes during abdominal infection (6, 12). FRCs constantly sense and monitor Toll-like receptor (TLR)-signaling ligands to regulate immune cell recruitment and survival within FALC. We have shown that activation of TLR9 signaling suppresses the production of chemokines and RA in FRCs, and thus reduces immune cell recruitment and peritoneal resident macrophage (PRM) retention during abdominal infection (4, 6). However, the roles of TLR9 signaling in regulating the population-specific functions of FRC are unclear, and distinct FRC subsets may have varying responses to TLR9 signaling.

In this study, we sought to study how TLR9 signaling regulates the functions of FRC subsets in the FALC to orchestrate peritoneal immunity against infection. Combining single-cell RNA sequencing (scRNAseq) and flow cytometry, we identified two FRC subsets, including CD55^hi^ and CD55^lo^ subsets in the murine mesenteric FALC and in human adipose tissues. Activation of TLR9 signaling suppressed the proliferation, chemokine production, and retinoid metabolism in only the CD55^lo^ subset. These data advance our understanding of the biology of FRC subsets in adipose tissues, which may be exploited for new strategies to treat peritoneal infection.

## 2 Materials and methods

### 2.1 Reagent

LPS-EB (tlrl-eblps), CpG ODN 1585 (tlrl-1585), CpG ODN 2216 (, tlrl-2216) were purchased from InvivoGen.

### 2.2 Mice

WT C57BL/6J mice, were obtained from the Jackson laboratory. *Tlr9^fl/fl^* mice on the C57BL/6J background were obtained from Mark J.Shlomchik lab (Department of Immunology, University of Pittsburgh) (13). Heterozygous BAC-transgenic C57BL/6N-Tg (Ccl19-Cre)489Biat (Ccl19-Cre) mice (*Ccl19^cre^*) mice were obtained from Dr. Burkhard Ludewig (Institute of Immunobiology, Kantonsspital St. Gallen, St. Gallen, Switzerland) (14, 15). FRC-specific *Tlr9* ^-/-^ (*Ccl19^Cre^Tlr9^fl/fl^*) mice were generated by cross-breeding with the *Tlr9^fl/fl^* and *Ccl19^cre^* mice. The genotype of *Ccl19^Cre^Tlr9^fl/fl^* mice were confirmed by PCR polymerase chain reaction (PCR)-based genotyping using multiple primer pairs, as described previously (4). These animals were bred in our facility. All mice were maintained under pathogen-free conditions. Mice were randomly assigned to different experimental or control groups between 6 and 8 weeks of age. All mice were acclimated in our animal facility for 3 or more days before any experiments.

All animal studies were approved by the Institutional Animal Care and Use Committees of the University of Pittsburgh and the Animal Care and Use Committee of The Feinstein Institute for Medical Research. Experiments were performed in adherence to the National Institutes of Health Guidelines.

### 2.3 Isolation of cells from mouse mesenteric FALC

Mice were sacrificed, and blood was extracted by cardiac puncture. Mesenteric adipose tissue was carefully excised from the small intestine, large intestine, and cecum using scissors without touching the pancreas or damaging the intestine. Mesenteric Lymph nodes were removed. Omentum and mesentery adipose tissue were minced in RPMI 1640 Medium (Gibco) containing 2% FBS (Biotechne), 60 μg/ml Liberase TL (millpore sigma), 250 μg/ml DNase I (millpore sigma). Minced adipose tissue was placed at 37°C for 25 minutes, shaking. Digestion was terminated by adding 4x volume RPMI 1640 with 10% FBS, and subsequently filtered through a 70 µm sterile filter. After centrifuging for 5 minutes at 250g, the supernatant was discarded. The cell pellet was resuspended with RPMI containing 5% FBS for further analysis.

### 2.4 Cecal ligation and puncture model

Mice that were 25 to 30 g in weight were used. The hair on the abdominal surface was removed. The abdominal skin was disinfected with a 2% iodine tincture. Laparotomy was performed under 2% isoflurane (Piramal Critical Care) with oxygen. For the sublethal model, 50% of the cecum was ligated and punctured twice with a 22-gauge needle. Saline (1 mL) was given subcutaneously for resuscitation immediately after the operation. Mice were sacrificed 18 hours after CLP to collect samples for assessing inflammatory index. For the lethal model, 75% of the cecum was ligated and punctured twice with an 18-gauge needle. Mice were monitored twice daily by personnel experienced in recognizing signs of a moribund state. Mice were euthanized with CO2 when they became moribund or at the observation endpoint (7 days). We used moribundity as the endpoint for our survival study following the Animal Research Advisory Committee Guidelines from NIH.

### 2.5 RNA extraction, cDNA synthesis, and quantitative PCR

Total RNA of cells was extracted using the RNeasy Mini Extraction Kit (QIAGEN) according to the manufacturer’s instructions. RNA quality and concentration are assessed using Bio Tek Synergy Mx and TAKE 3 plate system. Two-step, real-time reverse transcription PCR (RT-PCR) was performed as previously described (6) with forward and reverse primer pairs prevalidated and specific for indicated target genes (**Supplementary Table S1**). All samples were assayed in duplicate and normalized to *Gapdh* mRNA abundance.

### 2.6 Aldefluor assay

FALC cells were isolated as above. For *in vivo* experiments, the single cell suspension was resuspended in 1 mL Aldefluor assay buffer (STEMCELL) in FACS tubes. For *in vitro* experiments, 50,000 cells/ml FRCs were used. The reagent was prepared according to Aldefluor kit (STEMCELL) guidelines. Each sample has a quench tube and a test tube. DEAB buffer (5µL/sample) was aliquoted into the quench tube. Activated Aldefluor reagent (5µL/sample) was then added to 1mL single cell suspension in the test tube immediately. Cells were mixed by pipetting, and 0.5 mL of the mixture was transferred to quench tubes immediately. After 20 minutes incubation at 37°C, all tubes were centrifuged for 5 minutes at 250g, and the supernatant was discarded. Surface staining was performed as above before flow cytometry analysis.

### 2.7 Single-cell RNA sequencing

For *ex vivo* single-cell RNA sequencing, FALCs cells were isolated as described above, then subjected to single-cell cDNA library construction and sent for scRNA sequencing (10x Genomics). Approximately 5000 cells were targeted in each sample, and the final libraries were sequenced with pair-end on Illumina platform with at least 50,000 reads per cell. Raw FASTQ data were processed using the 10x Genomics CellRanger pipeline (v3.0.0). For alignment, reads were mapped to mouse genome mm10. Subsequent normalization, scaling and clustering were processed in R (version 3.6.0) using the Seurat package (version 3.0.0). The top feature genes of each cluster were used in pathway enrichment analysis by package clusterProfiler (version 4.0).

### 2.8 Flow cytometry

Cells were blocked for Fc receptors with anti-mouse CD16/32 (BD Bioscience) for 5 minutes. For surface staining, cells were incubated with fluorochrome-conjugated antibody (**Supplementary Table S2**) for 30 minutes at 4°C in dark. For intracellular staining, cells were fixed and permeabilized using the Foxp3/Transcription Factor Staining Buffer Set (eBioscience™), followed by incubation with fluorochrome-conjugated antibodies (**Supplementary Table S2**) for 30 minutes at 4°C in dark. Flow cytometry data were acquired using LSR II Flow Cytometer (Becton Dickinson) and LSRFortessa Flow Cytometer (Becton Dickinson) with FASCDiva Software (Version 8.0.1, BD Pharmingen) and analyzed with Flowjo software (version 10).

### 2.9 Adoptive transfer

FRCs were isolated from mesenteric adipose tissue. CD55^hi^ and CD55^lo^ FRCs were sorted as CD45-/CD31-/PDPN+/CD55^hi^ and CD45-/CD31-/PDPN+/CD55^lo^ using BD Aria Plus high-speed sorter. Sorted FRC were cultured and expanded in MesenCult Expansion full media (STEMCELL Technology) at 37°C with 5% CO2 for 7 days. FRCs (100000 cells in 200µL PBS) were resuspended in PBS and intraperitoneally injected into mice 4 hours after CLP.

### 2.10 Isolation of human FRCs

Human adipose tissue–derived stromal cells were obtained from the adipose stem cell center in the Department of Plastic Surgery at the University of Pittsburgh. The CD55^hi^ and CD55^lo^ human FRC were sorted (BD Aria Plus high-speed sorter) and identified as CD45^-^/CD31^-^/PDPN^+^/CD55^hi^ or CD45^-^/CD31^-^/PDPN^+^/CD55^lo^ cells respectively (**Supplementary Figures 1A-G**). Sorted human FRC subsets were cultured and expanded in MesenCult Expansion full media (STEMCELL Technology) at 37°C with 5% CO_2_ for 7 days. The purity of human FRCs subsets (CD45^-^/CD31^-^/PDPN^+^/CD55^hi^ or CD45^-^/CD31^-^/PDPN^+^/CD55^lo^) was assessed using flow cytometry (**Supplementary Figures 1H, I**).

### 2.11 Cell counting kit-8 proliferation assay

Human FRCs were plated in 96-well flat-bottom at a concentration of 2000 FRCs/well/100μL and cultured for 18 hours before being treated with ODN1585 (5 μM) or LPS (1 μg/mL) for the indicated time. Cell proliferation was evaluated using CCK-8 assay (Abcam) following manufactory’s instruction. The absorbance was measured at 460 nm using a plate reader.

### 2.12 Statistical analysis

Statistical analysis of quantitative PCR and quantitative results for flow cytometry was performed with Prism9 (GraphPad software, San Diego, USA). Unpaired, 2-tailed Student’s t tests were performed to compare 2 groups. One-way ANOVA or 2-way ANOVA with Bonferroni Correction were performed to compare 3 or more groups, as indicated in the figure legends. A p value of < 0.05 was considered statistically significant. All values are presented as mean ± SD. As for survival analysis, statistical differences were determined using the log-rank test.

## 3 Results

### 3.1 Specific deletion of TLR9 in FRCs improves outcomes in bacterial peritonitis

**Figure 1:**
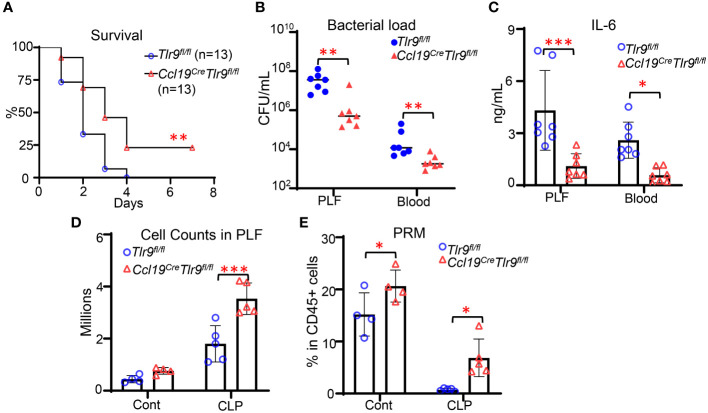
Specific deletion of TLR9 signaling in FRCs improves outcomes in bacterial peritonitis. **(A)** Kaplan-Meier survival curve. Data are pooled from 2 separate experiments. Statistical differences were determined using log-rank test adjusted by Bonferroni’s testing. **p < 0.01. **(B)** Bacterial load and **(C)** IL-6 levels in PLF and plasma, **(D)** Cell counts, and **(E)** the frequency of PRM in CD45+ cells in the PLF at 18 hours after CLP. Symbols represent individual mice. Data are shown as mean ± SD. Statistical differences were determined using two-way ANOVA adjusted by Bonferroni’s testing. *p < 0.05, **p < 0.01, ***p < 0.001.

We have previously shown that global deletion of TLR9 increases chemokines production and retinoid metabolism in FRCs from FALC, thus promoting immune cell recruitment and preventing peritoneal resident macrophage (PRM) disappearance in the peritoneal cavity (4, 6). Immune cell recruitment and PRMs are essential for effective host defense during peritoneal infection. Therefore, we hypothesized that specific deletion of TLR9 in FRCs would improve the outcome of bacterial peritonitis. To test this hypothesis, we generated the FRC-specific TLR9 knockout mice (*Ccl19^Cre^Tlr9^fl/fl^)* by crossing the *Tlr9^fl/fl^* mice (13) and *Ccl19^cre^* mice (14, 15). Our data indicated that they have similar phenotypes in terms of baseline expression of *Tlr9, Ccl19, Ccl21*, and *Il6* in FRC as well as bacterial clearance and IL6 levels in the plasma and bronchioalveolar lavage (BAL) at 18 hours after CLP (**Supplementary Figure 2**). Therefore, we will use the *Tlr9^fl/fl^* as the control of the *Ccl19^Cre^Tlr9^fl/fl^* mice in our study. *Ccl19^Cre^Tlr9^fl/fl^* and *Tlr9^fl/fl^* control mice were subjected to cecal ligation and punctures (CLP). As expected, specific deletion of *Tlr9* in FRCs reduced mortality, peritoneal bacterial load, as well as peritoneal and circulating IL-6 levels after CLP (**Figures 1A-C**). Of note, the improved outcomes in *Ccl19^Cre^Tlr9^fl/fl^* mice were associated with increased peritoneal cell (**Figure 1D**) and PRM (**Figure 1E**) numbers after CLP. These data demonstrate that the TLR9 signaling in FRC is critical for governing the peritoneal immunity during bacterial peritonitis.

### 3.2 CD55^lo^ FRC subsets in the mesenteric FALC are immunoregulatory

**Figure 2:**
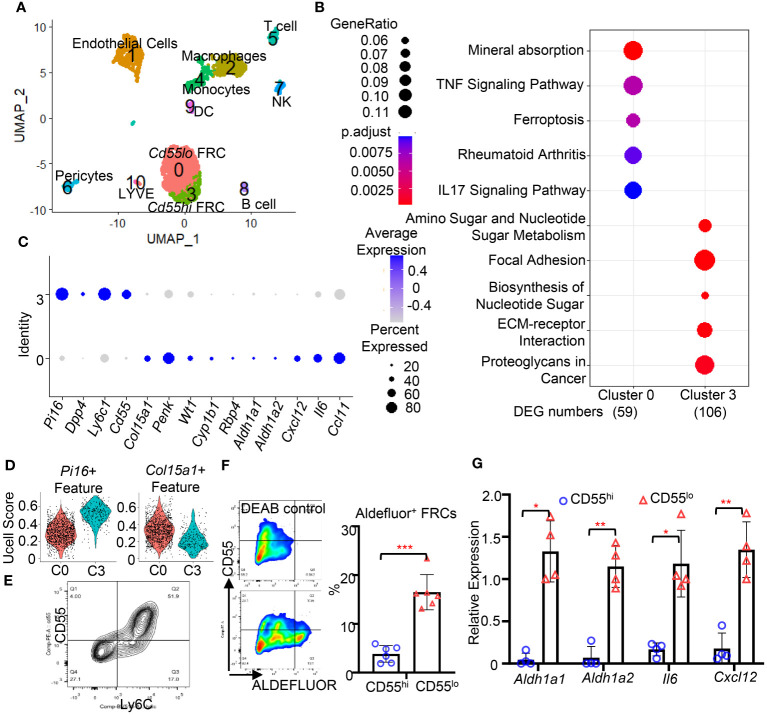
The CD55^lo^ FRC subset in the FALC is immunoregulatory. **(A)** A two-dimensional representation (UMAP) of global gene expression relationships among 2678 cells of mesenteric adipose tissue. **(B)** Dotplot of gene set enrichment analysis of cluster 0 and cluster 3. **(C)** Dotplot of indicated gene expression cluster 0 and cluster 3 FRCs. **(D)** Violin plot of signature score between FRC subsets and indicated fibroblast subsets. **(E)** Representative gating for CD55^hi^Ly6C^hi^ and CD55^lo^Ly6C^lo^ FRC subsets in WT mesenteric adipose tissue. **(F)** Representative gating and quantification of the frequency of Aldefluor^+^ FRC in WT mesenteric adipose tissue. **(G)** Expression levels of indicated genes in CD55^hi^ and CD55^lo^ FRC subsets in WT mesenteric adipose tissue. Data are shown as mean ± SD from 2 separate experiments. Symbols represent individual mice. Statistical differences were determined using 2-tailed Student’s t tests adjusted by Turkey correction. *p < 0.05, **p < 0.01, ***p <0.001.

FRCs are heterogeneous within organs and tissues. To understand the function of FRC subsets in the FALC, we performed scRNA-seq on cells of FALC isolated from the mesenteric adipose tissue in WT mice. Eleven cell clusters were identified and annotated according to their feature genes (**Figure 2A**). Two clusters of FRC (C0 & C3) were identified (**Figure 2A**). Performing differentially expressed gene (DEG) analysis (**Supplementary List 1**) and gene set enrichment analysis using the Kyoto Encyclopedia of Genes and Genomes (KEGG) pathway database (**Supplementary List 2**), we found that DEGs upregulated in C0 were enriched in gene expression related to immune responses. In contrast, DEGs upregulated in C3 were enriched in gene families related to extracellular matrix (ECM) formation (**Figure 2B**). Comparing the feature genes of these two FRC subsets with features of the fibroblast subset reported previously (11), we found that the C3 FRC shared similar gene expression as the *Pi16+* fibroblast subset (**Figures 2C, D**). The C0 FRC shared gene expressions similar to those of the *Col15a1+* fibroblast subset (**Figures 2C, D**). Interestingly, we found that the C0 subset highly expressed genes related to retinoid metabolism (Wt1, Cyp1b1, Rbp4, Aldh1a1, Aldh1a2) and immune response (Cxcl12, Il6, Ccl11, Ccl19) compared with the C3 subset (**Figure 2C**, **Supplementary Figures 2A, B**). Using flow cytometry detecting the expression of cell surface expressions of feature genes, CD55 and Ly6C (protein name for *Ly6c1*), we identified the CD55^hi^Ly6C^hi^ and CD55^lo^Ly6C^lo^ subsets of FRCs in the FALC of mouse mesenteric adipose tissue (**Figure 2E**), representing the C3 and C0 subsets identified by scRNAseq, respectively. CD55, the complement decay-accelerating factor expressed on the cell surface in both mice and humans (16). Therefore, we used CD55 as the marker to differentiate and sort these two FRC subsets from mouse and human adipose tissue. Using flow cytometry to analyze the ALDH activities for retinoid acid metabolism in the CD55^hi^ and CD55^lo^ subsets of FRC from the FALC, we found that the majority of Aldefluor^+^ FRC expressed low levels of CD55 (**Figure 2F**). Furthermore, we sorted the CD55^hi^ and CD55^lo^ subsets of FRC to assess the gene expression levels using qPCR. We found that the CD55^lo^ FRC subset expressed substantially higher levels of retinoid metabolic genes (*Aldh1a1 & Aldh1a2*) and inflammatory genes (*Il6 & Cxcl12*) compared with the CD55^hi^ FRC subsets (**Figure 2G**). These data indicate that the CD55^lo^ FRC subset is the immunoregulatory subset of the mesenteric FALC.

### 3.3 Activation of TLR9 signaling suppressed the proliferation of CD55^lo^ FRCs in the FALC

**Figure 3:**
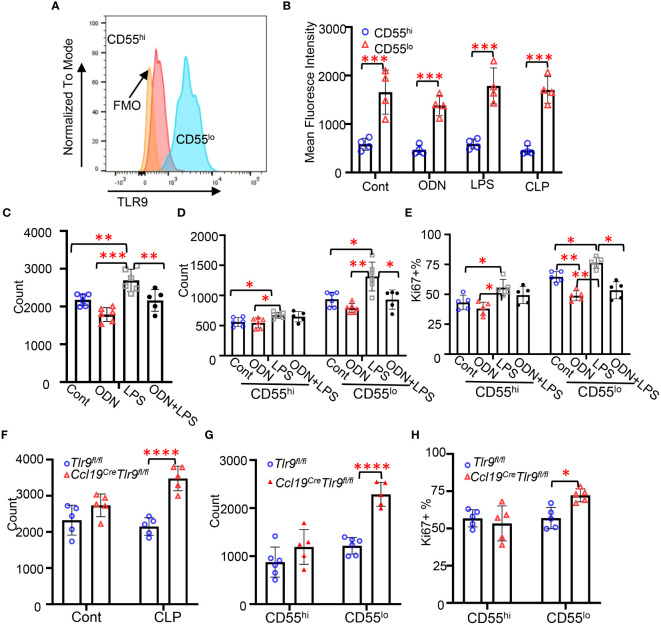
Activation of TLR9 signaling suppressed the proliferation of CD55lo FRCs in the FALC. **(A)** Representative gating of TLR9 expression in FRC subsets. **(B-E)** WT mice were intraperitoneally treated with PBS (Cont), ODN1585 (ODN, 2.5 nmol/mouse, LPS (5mg/kg), or ODN+LPS for 18 hours. **(B)** Mean fluorescence intensity of TLR9 in FRC subsets. **(C)** Numbers of FRCs, **(D)** Numbers of CD55^hi^ and CD55^lo^ FRCs, and **(E)** The frequency of Ki67+ FRC in the CD55^hi^ and CD55^lo^ subset in the mesenteric adipose tissue. **(F-H)** *Tlr9fl/fl* and *Ccl19creTlr9fl/fl* mice were subjected to CLP for 18h. **(F)** Numbers of FRCs, **(G)** Numbers of CD55^hi^ and CD55^lo^ FRCs, and **(H)** The frequency of Ki67+ FRC in the CD55^hi^ and CD55^lo^ subset in the mesenteric adipose tissue. Data are pooled from 2 separate experiments. Data are shown as mean ± SD. Symbols represent individual mice. Statistical differences were determined using two-way ANOVA adjusted by Bonferroni’s testing. *p < 0.05, **p < 0.01, ***p < 0.001, ****p < 0.0001.

TLR4 and TLR9 are major receptors sensing pathogen-associated molecular patterns (PAMP) and damage-associated molecular patterns (DAMP) to regulate peritoneal immunity during CLP-induced peritonitis (6, 17, 18). We have previously demonstrated that TLR9 plays a dominant role in regulating chemokine production in FRC during abdominal sepsis (6). To understand the roles of TLR9 in these FRC subsets during peritonitis, we challenged the mice with ODN (a TLR9 agonist, 2.5 nmol/mouse, i.p.), LPS (a TLR4 agonist, 5mg/kg, i.p.), and CLP for 18 hours. We first assessed the expression levels of TLR9 in these subsets using flow cytometry. We found that the expression levels of TLR9 in the CD55^lo^ FRC subset were substantially higher than the CD55^hi^ subset at baseline and in response to ODN, LPS, or CLP challenge (**Figures 3A, B**). Furthermore, we found that the number of FRCs and CD55^lo^ FRCs, but not the CD55^hi^ FRCs in the mesenteric adipose tissue slightly decreased after 18h ODN challenge (**Figures 3C, D**). The numbers of FRC, CD55^hi^, and CD55^lo^ FRCs increased in mice challenged with LPS (**Figures 3C, D**). The addition of ODN suppressed the LPS-induced increase in FRC and CD55^lo^ FRC numbers (**Figures 3C, D**), suggesting that ODN suppressed the proliferation of the CD55^lo^ FRCs. FRCs are proliferative for self-renewal (1). To test if ODN suppressed the proliferative ability of CD55^lo^ FRCs, we assessed the expression of the proliferative gene, ki67 using flow cytometry. As expected, treatment of ODN decreased the frequency of Ki67+ cell in the CD55^lo^ subset but not the CD55^hi^ subset in the mesenteric adipose tissues compared with the control (**Figure 3E**). In contrast, treatment with LPS increased the frequency of Ki67+ CD55^hi^ and CD55^lo^ FRCs (**Figure 3E**). The addition of ODN suppressed the LPS-induced increase in Ki67+ FRC in the CD55^lo^ subset but not the CD55^hi^ subset (**Figure 3E**). Of note, specific deletion of TLR9 in FRC increased the number and the frequency of Ki67+ FRCs in the CD55^lo^ subset but not the CD55^hi^ subset at 18h after CLP (**Figures 3F-H**). Together, these data indicate that TLR9 signaling is dominant in suppressing the proliferation of CD55^lo^ FRCs in the mesenteric FALC during inflammation.

### 3.4 Activation of TLR9 signaling suppressed the immunoregulatory functions of CD55^lo^ FRCs in the mesenteric FALC

**Figure 4:**
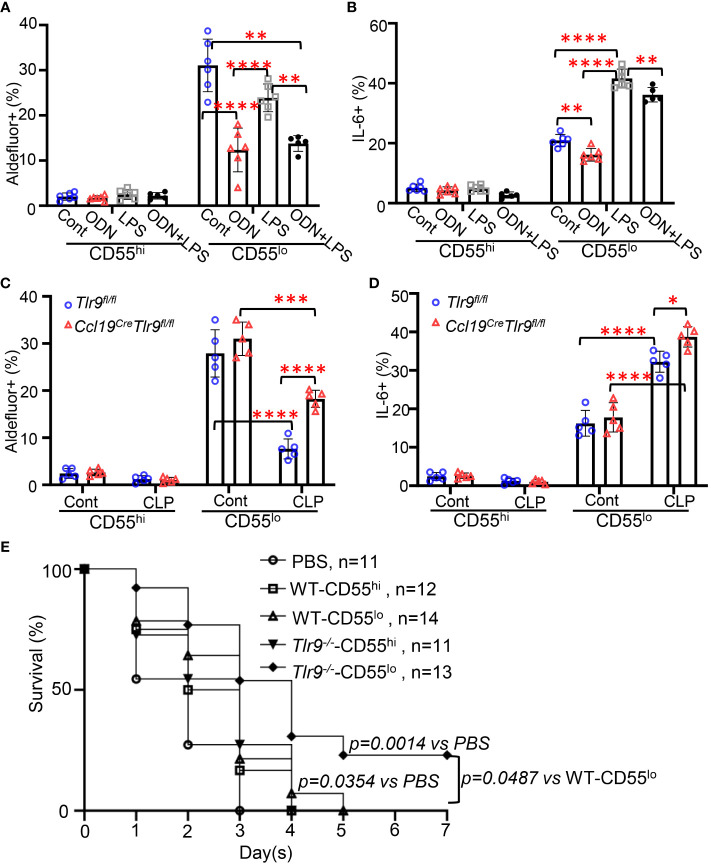
Activation of the TLR9 signaling suppressed the immunoregulatory functions of CD55lo FRCs in the FALC. **(A, B)** WT mice were intraperitoneally treated with PBS (Cont), ODN1585 (ODN, 2.5 nmol/mouse, LPS (5mg/kg), or ODN+LPS for 18 hours. **(A)** The frequency of Aldefluor+ FRCs and **(B)** The frequency of IL-6+ FRCs in the CD55^hi^ and CD55^lo^ subset in the mesenteric adipose tissue. **(C, D)** *Tlr9^fl/fl^* and *Ccl19^cre^Tlr9^fl/fl^* mice were subjected to CLP for 18h. **(C)** The frequency of Aldefluor+ FRCs and **(D)** The frequency of IL-6+ FRCs in the CD55^hi^ and CD55^lo^ subset in the mesenteric adipose tissue. **(E)** Kaplan-Meier survival curve of mice treated with PBS, WT-CD55^hi^-FRC, WT-CD55^lo^-FRC, *Tlr9* ^-/–^CD55^hi^-FRC, *Tlr9* ^-/–^CD55^lo^-FRC (100000 FRC/mouse, 50% of the previous dose) 4 hours after CLP. Data are pooled from 2 separate experiments. Statistical differences were determined using log-rank test adjusted by Bonferroni’s testing. Data are pooled from 2 separate experiments. Data are shown as mean ± SD. Symbols represent individual mice. Statistical differences were determined using two-way ANOVA adjusted by Bonferroni’s testing. *p < 0.05, **p < 0.01, ***p < 0.001, ****p < 0.0001.

We have previously shown that activation of TLR9 signaling suppresses chemokine production and RA metabolism, preventing PRM retention (4). Our data indicates that TLR9 and inflammatory genes are dominantly expressed in the CD55^lo^ FRC subset in the FALC (**Figures 3A, B**). To test the role of TLR9 signaling in the immunoregulatory functions in these FRC subsets, WT mice were treated with ODN, LPS, or ODN+LPS intraperitoneally for 18h. ODN treatment substantially decreased the frequency of Aldefluor+ and IL6+ FRC in the CD55^lo^ subset but not the CD55^hi^ subset in the mesenteric adipose tissue compared with control (**Figures 4A, B**, **Supplementary Figure 4**). The frequency of Aldefluor+ FRC was similar between LPS-treated mice and control mice in both subsets (**Figure 4A**). The frequency of IL6+ FRC substantially increased after LPS treatment compared with the control in the CD55^lo^ subset but not the CD55^hi^ subset (**Figure 4B**, **Supplementary Figure 4**). The addition of ODN substantially suppressed the LPS-induced increase in the frequency of Aldefluor+ and IL6+ CD55^lo^ FRC (**Figures 4A, B**, **Supplementary Figure 4**). Notable, the frequency of Aldefluor+ and IL6+ CD55^hi^ FRC remained at the low baseline level in response to ODN, LPS, or ODN+LPS stimulation (**Figures 4A, B**). Furthermore, specific deletion of TLR9 in FRC increased the frequency of Aldefluor+ and IL6+ CD55^lo^ FRC compared with the Flox control 18h after CLP (**Figures 4C, D**). The frequency of Aldefluor+ and IL6+ CD55^hi^ FRC was similar between the *Ccl19^Cre^Tlr9^fl/fl^* and the flox control at 18h after CLP (**Figures 4C, D**). We have previously shown that adoptive transfer of a low dose of *Tlr9* ^-/–^FRC (200000 FRC/mouse) improved the therapeutic efficacy in CLP-induced sepsis compared with the treatment with the same amount of WT-FRC (4, 6). To understand the role of TLR9 of these FRC subsets in the therapeutic efficacy of CLP-induced sepsis, we performed adoptive transfer experiments with these FRC subsets. Our data indicated that the CD55^hi^ or the CD55^lo^ FRCs are approximately 50% of total FRCs (**Figure 2E**). We treated the mice with PBS, WT-CD55^hi^-FRC, WT-CD55^lo^-FRC, *Tlr9* ^-/–^CD55^hi^-FRC, *Tlr9* ^-/–^CD55^lo^-FRC (100000 FRC/mouse, 50% of the previous dose) 4 hours after CLP. Notably, adoptive transfer of *Tlr9* ^-/–^CD55^lo^ FRCs improved 7-day survival after CLP (**Figure 4E**). These data indicate that the TLR9 signaling governs the immunoregulatory functions of the CD55^lo^ FRC subset.

### 3.5 TLR9 signaling suppresses the proliferation and immunoregulatory functions in the CD55^lo^ FRC subset from human adipose tissue

**Figure 5:**
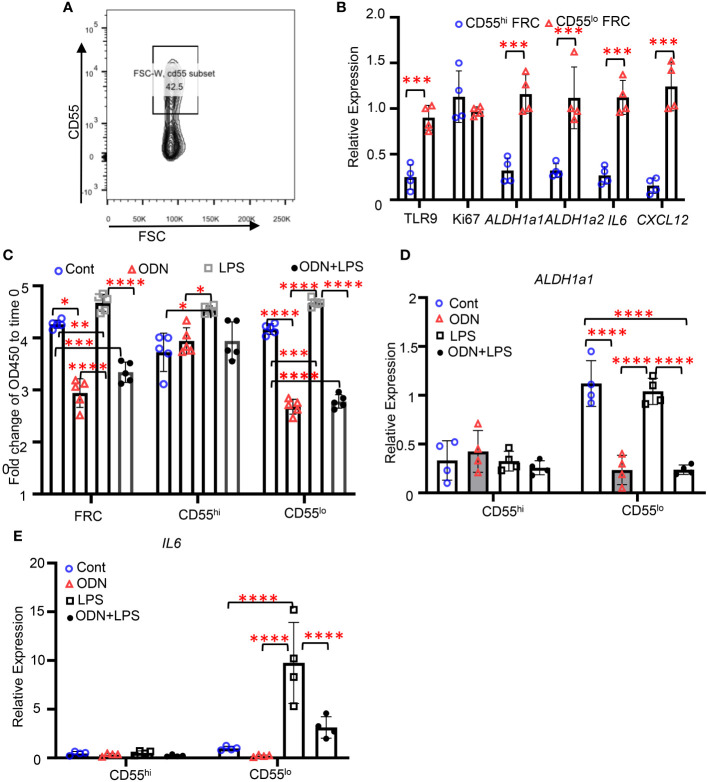
TLR9 signaling suppresses the proliferation and immunoregulatory functions in the CD55^lo^ FRC subset from human adipose tissue. **(A)** Representative gating for CD55^hi^ and CD55^lo^ FRC subsets in human adipose tissue. **(B-D)** CD55^hi^ and CD55^lo^ FRCs were sorted from human adipose tissue and treated with PBS (Cont), ODN1585 (ODN, 2.5nM, LPS (1μg/mL), or ODN+LPS for 18 hours. **(B)** Expression levels of indicated genes assessed using quantitative PCR. **(C)** Fold change of OD450 to time 0 of indicated cell subsets 96h after indicated treatments assessed using CCK8 assay. **(D)** Expression levels of ALDH1a1 in CD55^hi^ and CD55^lo^ FRCs after indicated treatments. **(E)** Expression levels of IL6 in CD55^hi^ and CD55^lo^ FRCs after indicated treatments. Data are shown as mean ± SD from a representative experiment. Statistical differences were determined using two-way ANOVA adjusted by Bonferroni’s testing. *p < 0.05, **p < 0.01, ***p < 0.001, ****p < 0.0001.

We next identified the CD55^hi^ and CD55^lo^ FRC subsets from lipoaspirates human adipose tissue (**Figure 5A**). Performing quantitative PCR with sorted CD55^hi^ and CD55^lo^ FRCs, we found that the expression levels of *TLR9*, *ALDH1a1*, *ALDH1a2*, *IL6*, and *CXCL12* were substantially higher in the CD55^lo^ subset than the CD55^hi^ subset at baseline (**Figure 5B**). There was no difference in the expression levels of Ki67 between these two subsets at baseline (**Figure 5B**). Using the CCK8 proliferative assay, we found that activating TLR9 with ODN suppressed the proliferation in the CD55^lo^ subset but not the CD55^hi^ subset (**Figure 5C**). Furthermore, treatment of ODN decreased the expression levels of *ALDH1a1* and *IL-6* in the CD55^lo^ FRCs but not the CD55^hi^ FRCs compared with the control (**Figure 5D**). Treatment of LPS increased the expression levels of IL6 in the CD55^lo^ FRCs but not the CD55^hi^ FRCs compared with the control (**Figure 5E**). Importantly, addition of ODN suppressed the LPS-induced increase in *IL6* expression in the CD55^lo^ FRCs (**Figure 5E**). These data show that the subset specific expression and function of TLR9 can be recapitulated in human FALC.

## 4 Discussion

In this study, using the FRC-specific TLR9 knockout mice, we confirmed that TLR9 signaling in FRC plays critical roles in peritoneal immunity for host defense. Furthermore, we found that activation of TLR9 suppresses genes required for proliferation, chemotaxis, and retinol synthesis in CD55^lo^ FRC but not CD55^hi^ FRC. Notably, we found that adoptive transfer of *Tlr9* ^-/-^ -Cd55^lo^- FRC derived from mesenteric FALC improved the therapeutic efficacy in CLP-induced peritonitis. Furthermore, we identified the CD55^hi^ and CD55^lo^ FRC subsets from human adipose tissue and validated the regulatory role of TLR9 in human CD55^lo^ FRCs derived from adipose tissue. These findings unravel the subset-specific regulatory role of TLR9 in FRC derived from adipose tissue, which may be targeted for precisely modulating the intra-peritoneal immune response.

Subsets of fibroblasts have been identified in lymphoid tissues, including the spleen (7) and LN (8). During development and homeostasis, fibroblast subsets in different lymphoid organ locations have niche-specific signatures and functions, which are critical for establishing the lymphoid structure. For instance, follicular dendritic cells at the follicle center and marginal reticular cells at the edge of the follicle adjacent to the subcapsular sinus express the chemokine CXCL13 and guide B cells to LN follicles (19, 20). FRCs in the T cell zone attract CCR7+ lymphocytes (21) and produce trophic factors such as interleukin-7 (22). Unlike the spleen and LN, FALC is the tertiary lymphoid tissue residing in the adipose tissue without classical lymphoid structure (23). Instead of the reported niche-specific FRC subsets in the well-organized secondary lymphoid organs, we identified two FRC subsets, the CD55^hi^ and CD55^lo^ subsets, in the FALC from the mesenteric adipose tissue during homeostasis. CD55 is the complement decay-accelerating factor expressed on the cell surface of mouse and human fibroblasts (11). We found that the CD55^hi^ subset is enriched in gene expression related to ECM formation, whereas the CD55^lo^ subset is enriched in gene expression for immune response in mouse and human FRCs. This is consistent with a previous study that identified these two subsets in the human epidermal adipose tissue and demonstrated that CD55^hi^ stromal cells are responsible for wound healing (24).

Using flow cytometry, we identified the CD55^hi^ and CD55^lo^ subsets in FALC after TLR challenges or CLP. Furthermore, we observed that the ratio of CD55^hi^/CD55^lo^ FRC in the FALC changed in response to TLR stimulations (**Supplementary Figure 4**). Multiple possibilities may contribute to this change. First, the cell proliferative abilities differ between the CD55^hi^ and CD55^lo^ subsets in response to TLRs. Similar to previous studies (25, 26), we found that activation of TLR4 signaling enhanced the proliferation of FRCs. Furthermore, LPS induced proliferation in both CD55^hi^ and CD55^lo^ FRC subsets. In contrast, activation of TLR9 suppressed the proliferation of CD55^lo^ FRCs but not CD55^hi^ FRCs, consisting of the differential expression of TLR9 between these two subsets. Secondly, new subsets of fibroblast reticular cells may emerge. Previous studies have reported that new subsets of fibroblast reticular cells emerge and support the induction and regulation of innate and adaptive immune processes in specific lymphoid niches during inflammation (8, 27, 28). However, with the limited resolution power of flow cytometry, we did not identify any new subsets of FRC in FALC. Future studies employing single-cell technologies, which have higher resolution power, are needed to understand if new subsets of FRCs emerge in the FALC within the peritoneal cavity during inflammation. Furthermore, according to the fibroblast atlases from humans and mice (11), the CD55^hi^ subset we identified shared similar gene expression profile with the Pi16+ universal fibroblast subset and the CD55^lo^ FRC shared a gene expression profile similar to the Col15a1+ universal fibroblast subset. Trajectory analysis predicts that fibroblasts emerge from Pi16+ universal fibroblasts, pass through a Col15a1+ universal fibroblast phase, and then terminally differentiate as specialized fibroblasts (11). It remains unclear whether the Col15a1+ CD55^lo^ subset differentiates from the Pi16+ CD55^hi^ subset in FALC and whether or how TLR9 signaling regulates the differentiation during homeostasis and inflammation. Future studies employing lineage tracing and fate mapping studies will be required to truly understand the relations between these two subsets in homeostasis and response to inflammation.

FRCs in the FALC sense the pathogen recognition patterns (PAMP) and damage association patterns (DAMP) through TLRs, enabling a rapid change in FALC composition in response to pathogen invasion (14, 29). FRCs in FALC have been shown to drive protective immunity in response to *Salmonella* infection through interactions with monocytes and B cells in the peritoneal cavity (14). TLR2 and TLR4 signaling plays a role in regulating the immunomodulatory functions of FRCs, enabling these cells to orchestrate peritoneal immunity in mouse *Salmonella* infection (14). TLR9 is an endosomal DNA sensor sensing exogenous or endogenous unmethylated CpG DNA to initiate inflammatory responses. We have previously shown that activation of TLR9 signaling regulates peritoneal immunity via decreasing chemokine production for immune cell recruitment and suppressing retinoid metabolism in FRCs, which is essential for PRM retention (4, 6). In this study, we found that TLR9 is dominantly expressed in the CD55^lo^ FRC subset. The expression levels of TLR9 in the CD55^lo^ FRC subset remained at the baseline level in response to the LPS or ODN challenge. Activation of TLR9 signaling dominantly suppressed the proliferation, chemokine production, and retinoid metabolism in the CD55^lo^ FRC subset. In contrast, activation of the TLR4 signaling with LPS increased the proliferation of both FRC subsets. Furthermore, the addition of TLR9 agonist suppressed the LPS-induced increase of proliferation and chemokine production in the CD55^lo^ subset. These results demonstrate that the TLR9 signaling controls the immune response in the CD55^lo^ FRC subset. However, further studies are required to clarify whether other TLRs are differentially expressed between these two subsets and the role of other TLRs in regulating the pathobiology of these FRC populations.

In summary, we have provided compelling evidence showing a dominant role for TLR9 in regulating the proliferation and immunoregulatory function in the CD55^lo^ FRC subset in mouse mesenteric FALC and human adipose tissue. Given that FRC-based therapies have shown promising therapeutic efficacy in models of peritoneal infections (6, 12), insights from these studies will provide the framework to standardize and improve FRC-based therapies for peritonitis.

## Data availability statement

The data presented in the study are deposited in the Gene Expression Omnibus (GEO) repository, accession number GSE 250571.

## Ethics statement

The studies involving humans were approved by University of Pittsburgh Institutional Review Board. The studies were conducted in accordance with the local legislation and institutional requirements. The human samples used in this study were acquired from gifted from another research group. Written informed consent for participation was not required from the participants or the participants’ legal guardians/next of kin in accordance with the national legislation and institutional requirements. The animal study was approved by Institutional Animal Care and Use Committees of the University of Pittsburgh the Animal Care and Use Committee of The Feinstein Institute for Medical Research. The study was conducted in accordance with the local legislation and institutional requirements.

## Author contributions

TJ: Investigation, Writing – original draft, Writing – review & editing, Data curation, Formal Analysis, Methodology, Validation. YL: Data curation, Formal Analysis, Methodology, Validation, Writing – review & editing. XH: Data curation, Formal Analysis, Software, Writing – review & editing. PJ: Data curation, Methodology, Writing – review & editing. TB: Conceptualization, Funding acquisition, Resources, Supervision, Writing – review & editing. MD: Conceptualization, Funding acquisition, Investigation, Project administration, Resources, Supervision, Writing – original draft, Writing – review & editing.

## Supplementary material

Supplementary Table 1 PCR primer information.

Supplementary Table 2 Flow antibody information.
