# Supplementary material for: Activation of TLR9 signaling suppresses the immunomodulating functions of CD55lo fibroblastic reticular cells during bacterial peritonitis
Source: Front Immunol. 2024 May 17;15:1337384. doi: 10.3389/fimmu.2024.1337384 (PMC11140099; doi:10.3389/fimmu.2024.1337384)
Supplement: Supplementary Table 1 — PCR primer information. [file Table_1.docx]

**Table S1 PCR primer information**

| Name | Sequence/description | |
| --- | --- | --- |
| Ms_Aldh1a1 | Forward Primer | ATACTTGTCGGATTTAGGAGGCT |
|  | Reverse Primer | GGGCCTATCTTCCAAATGAACA |
| Ms_Aldh1a2 | Forward Primer | CAGAGAGTGGGAGAGTGTTCC |
|  | Reverse Primer | CACACAGAACCAAGAGAGAAGG |
| Ms_cxcl12 | Product Name: Mm_Cxcl12_1_SG QuantiTect Primer Assay  GeneGlobe Id: QT00161112  Catalog Number: 249900 | |
| Ms_IL6 | Forward Primer | TAGTCCTTCCTACCCCAATTTCC |
|  | Reverse Primer | TTGGTCCTTAGCCACTCCTTC |
| H_GAPDH | Forward Primer | GGAGCGAGATCCCTCCAAAAT |
|  | Reverse Primer | GGCTGTTGTCATACTTCTCATGG |
| H_Aldh1a1 | Forward Primer | GCCGGAGGAAATGTACCAGAC |
|  | Reverse Primer | CCCCTTGAAGGTAGGGCAG |
| H_ Aldh1a2 | Product Name: Hs_ALDH1A2_1_SG QuantiTect Primer Assay  GeneGlobe Id: QT00066045  Catalog Number: 249900 | |
| H_Ki67 | Product Name: Hs_MKI67_1_SG QuantiTect Primer Assay  GeneGlobe Id: QT00014203  Catalog Number: 249900 | |
| H_cxcl12 | Product Name: Hs_CXCL12_1_SG QuantiTect Primer Assay  GeneGlobe Id: QT00087591  Catalog Number: 249900 | |
| H_IL6 | Product Name: Hs_IL6_1_SG QuantiTect Primer Assay  GeneGlobe Id: QT00083720  Catalog Number: 249900 | |
| H_TLR9 | Product Name: Hs_TLR9_va.1_SG QuantiTect Primer Assay  GeneGlobe Id: QT02449265  Catalog Number: 249900 | |
