# Supplementary material for: Activation of TLR9 signaling suppresses the immunomodulating functions of CD55lo fibroblastic reticular cells during bacterial peritonitis
Source: Front Immunol. 2024 May 17;15:1337384. doi: 10.3389/fimmu.2024.1337384 (PMC11140099; doi:10.3389/fimmu.2024.1337384)
Supplement: Supplementary Table 2 — Flow antibody information. [file Table_2.docx]

Table S2. Flow antibody information

| antibody | clone | company |
| --- | --- | --- |
| FRC staining （mouse） | | |
| BUV395 Rat Anti-Mouse CD45 | 30-F11 | BD Biosciences |
| BUV395 Rat Anti-Mouse CD31 | 390 | BD Biosciences |
| PE-Cy™7 Rat Anti-Mouse CD31 | 390 | BD Biosciences |
| Brilliant Violet 421™ anti-mouse CD140a | APA5 | BioLegend |
| APC anti-mouse Podoplanin | 8.1.1 | BioLegend |
| PE anti-mouse CD55 (DAF) | RIKO-3 | BioLegend |
| PerCP/Cyanine5.5 anti-mouse CD9 | MZ3 | BioLegend |
| FITC anti-mouse CD26 (DPP-4) | H194-112 | BioLegend |
| FRC staining （human） | | |
| APC-Cy™7 Mouse Anti-Human CD31 | WM59 | BD Biosciences |
| APC-Cy™7 Mouse Anti-Human CD45 | 2D1 | BD Biosciences |
| PE-Cy™5 Mouse Anti-Human CD45 | HI30 | BD Biosciences |
| BUV395 Mouse Anti-Human Podoplanin | LpMab-23 | BD Biosciences |
| PerCP-Cy™5.5 Mouse Anti-Human CD140a | αR1 | BD Biosciences |
| APC Mouse Anti-Human CD55 | IA10 | BD Biosciences |
| Immune cells staining | | |
| PE/Cyanine7 anti-mouse/human CD11b | M1/70 | BioLegend |
| APC/Cyanine7 anti-mouse/human CD11b | M1/70 | BioLegend |
| PerCP/Cy5.5-anti-mouse CD11b | M1/70 | BioLegend |
| PE/Cyanine7 anti-mouse I-A/I-E （MHC II） | M5/114.15.2 | BioLegend |
| Brilliant Violet 605™ anti-mouse F4/80 | BM8 | BioLegend |
| Brilliant Violet 421 anti-mouse F4/80 | BM8 | BioLegend |
| Alexa Fluor® 700 anti-mouse CD11c | N418 | BioLegend |
| PE-Cy-7 Rat Anti-mouse CD45 | 30-F11 | BD Biosciences |
| BUV395 Rat Anti-mouse Ly-6G | 1A8 | BD Biosciences |
| Alexa Fluor 700 Rat Anti-mouse CD3 | 17A2 | BioLegend |
| PerCP/Cy5.5-anti-mouse CD4 | GK1.5 | BioLegend |
| V450 anti-mouse CD8a | 53-6.7 | eBioscience |
| Fixable Viability Dye eFluor™ 780 |  | eBioscience |
| Intracellular staining | | |
| PE Mouse Anti-Mouse CD289 (TLR9) | J15A7 | BD Biosciences |
| Alexa Fluor™ 488 anti-mouse/human Ki-67 | SolA15 | eBioscience |
| Human/Mouse CXCL12/SDF-1 PE-conjugated Antibody | 79018 | R&D System |
