# Supplementary material for: Activation of TLR9 signaling suppresses the immunomodulating functions of CD55lo fibroblastic reticular cells during bacterial peritonitis
Source: Front Immunol. 2024 May 17;15:1337384. doi: 10.3389/fimmu.2024.1337384 (PMC11140099; doi:10.3389/fimmu.2024.1337384)
Supplement: Supplementary file 5 [file Presentation_1.pptx]

## Slide 1
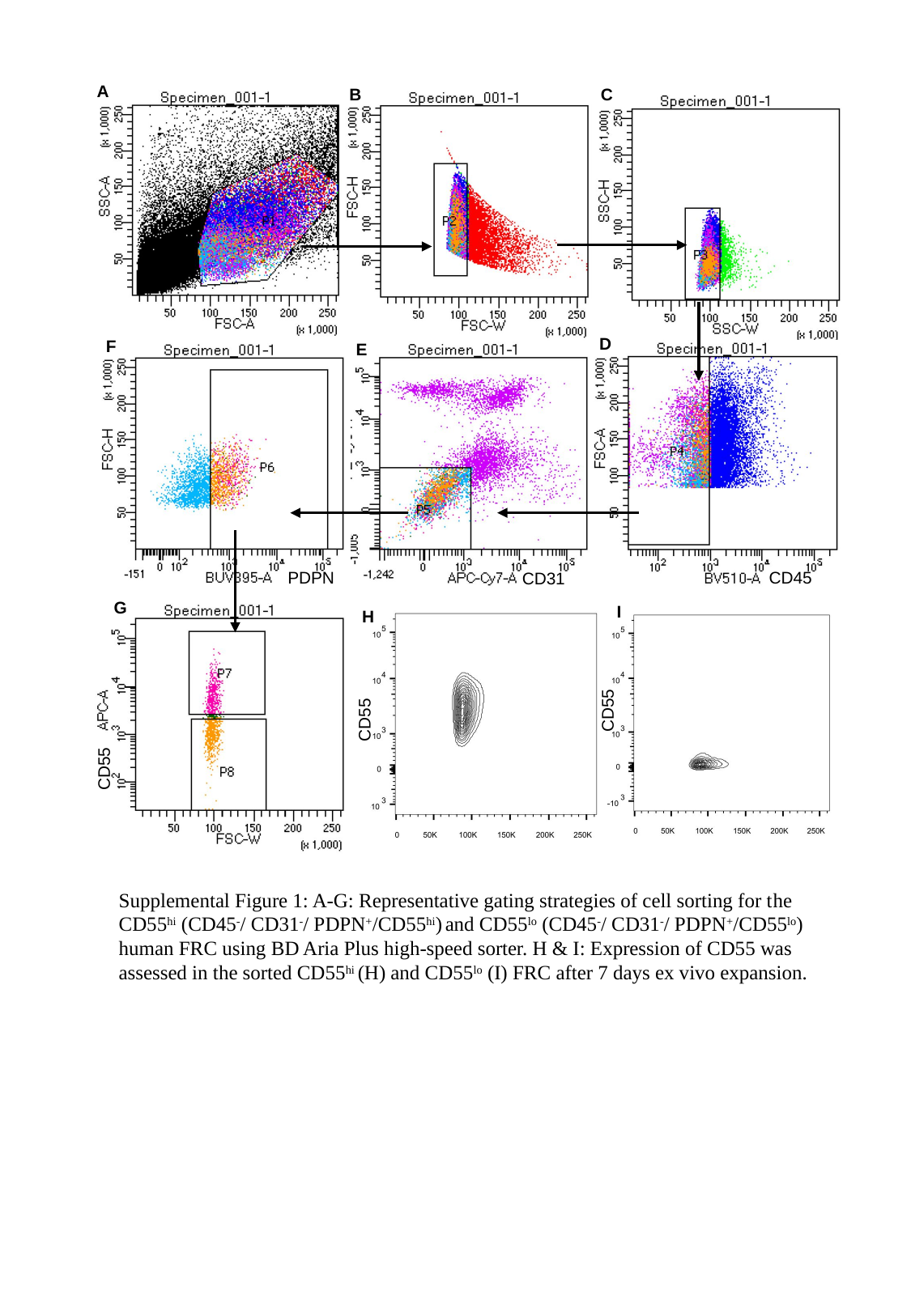

A
B
C
D
F
E
PDPN
CD45
CD31
G
I
H
CD55
CD55
CD55
Supplemental Figure 1: A-G: Representative gating strategies of cell sorting for the CD55hi (CD45-/ CD31-/ PDPN+/CD55hi) and CD55lo (CD45-/ CD31-/ PDPN+/CD55lo) human FRC using BD Aria Plus high-speed sorter. H & I: Expression of CD55 was assessed in the sorted CD55hi (H) and CD55lo (I) FRC after 7 days ex vivo expansion.

## Slide 2
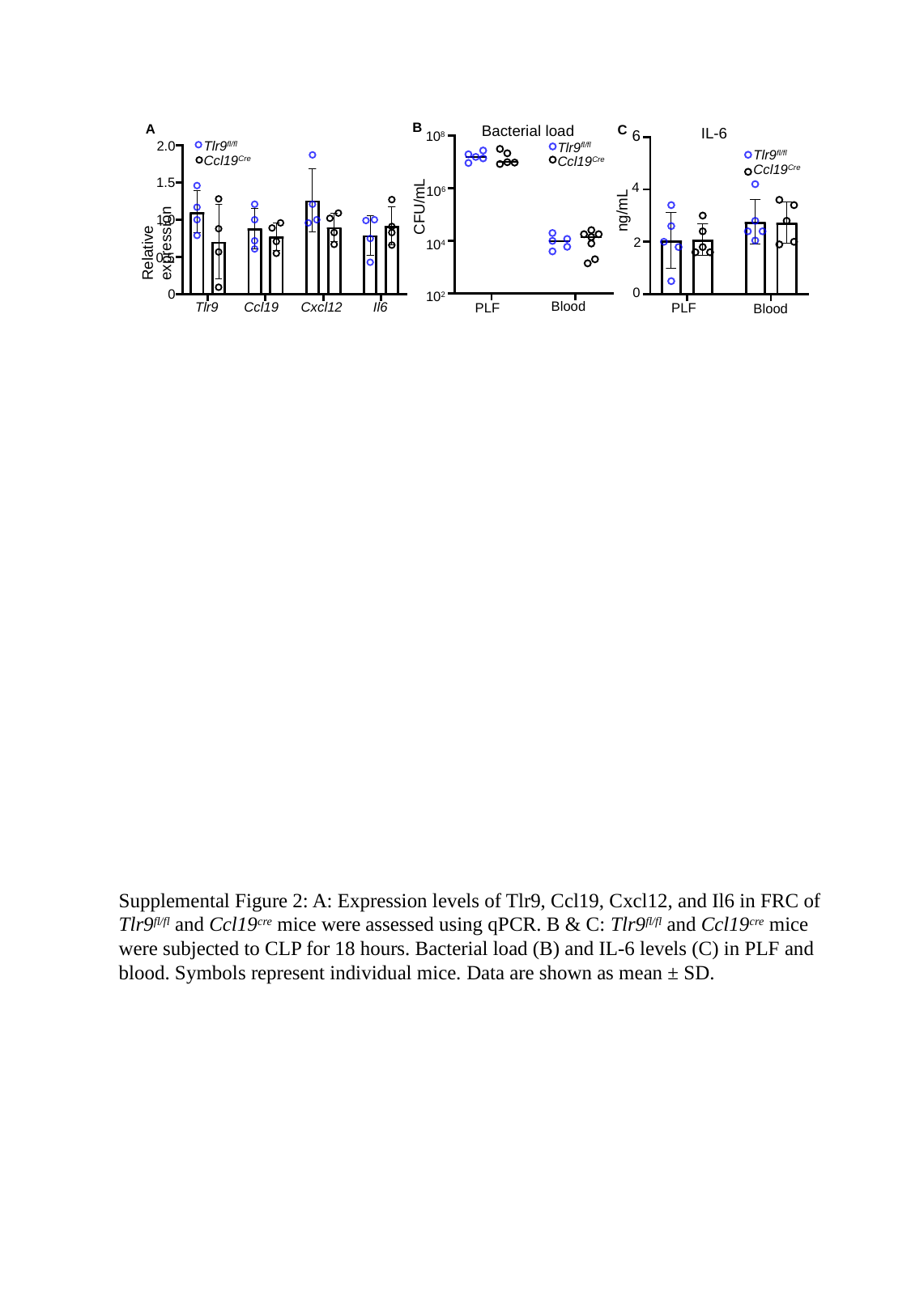

B
A
2.0
Tlr9fl/fl
Ccl19Cre
1.5
Relative expression
1.0
0.5
0
Tlr9
Ccl19
Cxcl12
Il6
C
IL-6
6
Tlr9fl/fl
Ccl19Cre
4
ng/mL
2
0
PLF
Blood
Bacterial load
108
Tlr9fl/fl
Ccl19Cre
CFU/mL
106
104
102
Blood
PLF
Supplemental Figure 2: A: Expression levels of Tlr9, Ccl19, Cxcl12, and Il6 in FRC of Tlr9fl/fl and Ccl19cre mice were assessed using qPCR. B & C: Tlr9fl/fl and Ccl19cre mice were subjected to CLP for 18 hours. Bacterial load (B) and IL-6 levels (C) in PLF and blood. Symbols represent individual mice. Data are shown as mean ± SD.

## Slide 3
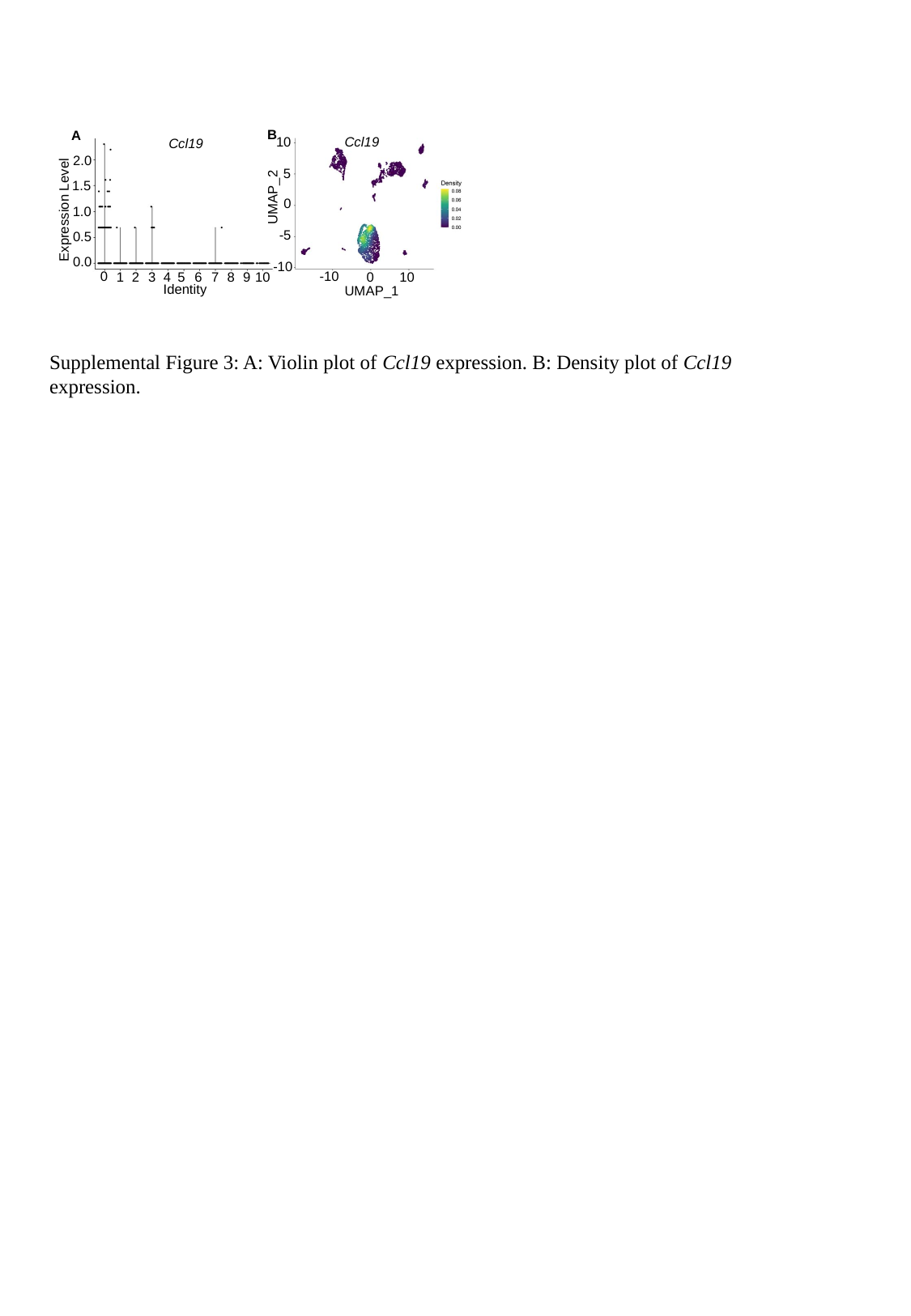

B
A
Ccl19
10
Ccl19
2.0
5
1.5
UMAP_2
0
Expression Level
1.0
-5
0.5
0.0
-10
0
-10
1
9
10
3
4
5
6
7
8
10
0
2
Identity
UMAP_1
Supplemental Figure 3: A: Violin plot of Ccl19 expression. B: Density plot of Ccl19 expression.

## Slide 4
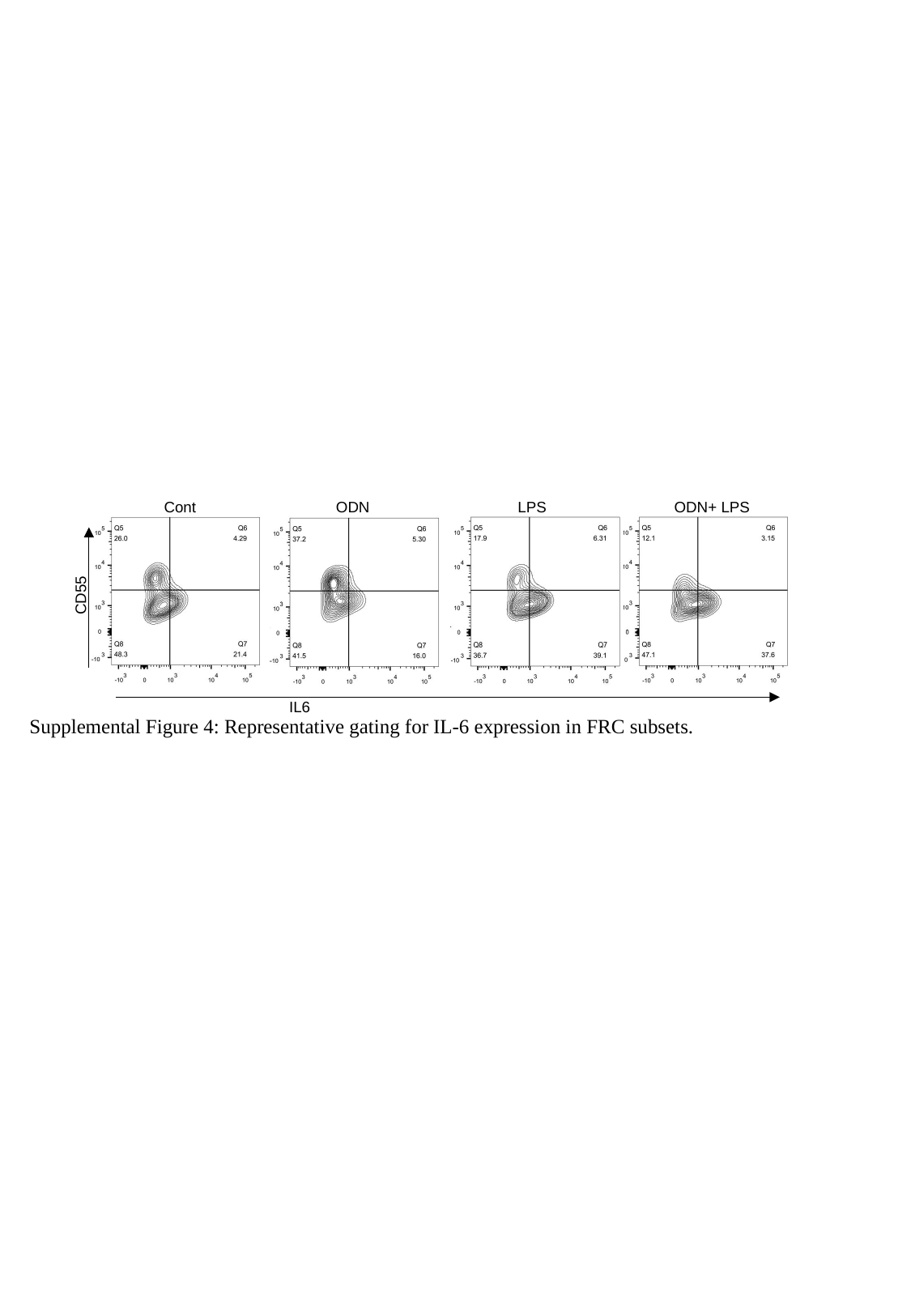

Cont
ODN
LPS
ODN+ LPS
CD55
IL6
Supplemental Figure 4: Representative gating for IL-6 expression in FRC subsets.
